# The Florida Clinical Skills Collaborative: A New Regional Consortium for the Assessment of Clinical Skills

**DOI:** 10.7759/cureus.31263

**Published:** 2022-11-08

**Authors:** Rebecca Toonkel, Analia Castiglioni, Debra Danforth, Lauren Fine, Jennifer Foster, Mario Jacomino, Michelle Johnson, Bridget Keller, Paul Mendez, John M Saunders, Ross Scalese, Dawn M Schocken, Carolyn Stalvey, Maria Stevens, Niharika Suchak, Samantha Syms, Emiri Uchiyama, Maria Velazquez

**Affiliations:** 1 Department of Medical Education and Simulation, Florida International University, Herbert Wertheim College of Medicine, Miami, USA; 2 Department of Medical Education and Simulation, University of Central Florida College of Medicine, Orlando, USA; 3 Department of Medical Education and Simulation, Florida State University College of Medicine, Tallahassee, USA; 4 College of Allopathic Medicine, Nova Southeastern University Dr. Kiran C. Patel College of Allopathic Medicine, Fort Lauderdale, USA; 5 Department of Internal Medicine, Florida Atlantic University Charles E. Schmidt College of Medicine, Boca Raton, USA; 6 Department of Pediatrics, Florida Atlantic University Charles E. Schmidt College of Medicine, Boca Raton, USA; 7 Department of Medical Education and Simulation, Nova Southeastern University Dr. Kiran C. Patel College of Osteopathic Medicine, Fort Lauderdale, USA; 8 Department of Neurology, Lake Erie College of Osteopathic Medicine, Bradenton, USA; 9 Department of Medical Education and Simulation, University of Miami Miller School of Medicine, Miami, USA; 10 Department of Pediatric Medicine, Florida International University, Herbert Wertheim College of Medicine, Miami, USA; 11 Department of Medical Education, University of South Florida (USF) Health Morsani College of Medicine, Tampa, USA; 12 Department of Medical Education and Simulation, University of Florida College of Medicine, Gainesville, USA; 13 Department of Humanities, Health, and Society, Florida International University, Herbert Wertheim College of Medicine, Miami, USA

**Keywords:** complex 2 pe, step 2 cs, consortia, osce, assessment, clinical skills

## Abstract

Discontinuation of the United States Medical Licensing Examination (USMLE) Step 2 Clinical Skills (CS) exam and Comprehensive Osteopathic Medical Licensing Examination (COMLEX) Level 2 Performance Evaluation (2-PE) raised questions about the ability of medical schools to ensure the clinical skills competence of graduating students. In February 2021, representatives from all Florida, United States, allopathic and osteopathic schools initiated a collaboration to address this critically important issue in the evolving landscape of medical education. A 5-point Likert scale survey of all members (n=18/20 individuals representing 10/10 institutions) reveals that initial interest in joining the collaboration was high among both individuals (mean 4.78, SD 0.43) and institutions (mean 4.69, SD 0.48). Most individuals (mean 4.78, SD 0.55) and institutions (mean 4.53, SD 0.72) are highly satisfied with their decision to join. Members most commonly cited a “desire to establish a shared assessment in place of Step 2 CS/2-PE” as their most important reason for joining. Experienced benefits of membership were ranked as the following: 1) Networking, 2) Shared resources for curriculum implementation, 3) Scholarship, and 4) Work towards a shared assessment in place of Step 2 CS/2-PE. Challenges of membership were ranked as the following: 1) Logistics such as scheduling and technology, 2) Agreement on common goals, 3) Total time commitment, and 4) Large group size. Members cited the “administration of a joint assessment pilot” as the highest priority for the coming year. Florida has successfully launched a regional consortium for the assessment of clinical skills competency with high levels of member satisfaction which may serve as a model for future regional consortia.

## Introduction

In January 2021, citing ‘the rapidly evolving medical education, practice and technology landscapes,’ the National Board of Medical Examiners (NBME) announced the discontinuation of the Step 2 Clinical Skills (CS) portion of the United States Medical Licensing Exam (USMLE). Discontinuation of the Comprehensive Osteopathic Medical Licensing Examination (COMLEX) Level 2 Performance Evaluation (2-PE) followed shortly thereafter [1,2,3]. Many valid concerns about the cost, convenience, and value of these exams had been raised over the years [4,5]. In fact, prior to discontinuation, the Step 2 CS exam was suspended temporarily in May 2020 during the COVID-19 pandemic with the goal of relaunching a modified exam to address many of these criticisms. 

While most of the concerns were legitimate, the presence of these exams undoubtedly drove institutional and learner support of CS teaching in the United States. [6-8]. The exams were not perfect, but they did provide generalizable psychometric-based national standards [9]. It is clear that assessment drives learning and the absence of these exams has brought to light the critical importance of ensuring continued standards of clinical skills competence. 

The Association of American Medical Colleges’ (AAMCs') Group on Educational Affairs (GEA) recently announced a new Constituent Collaborative Project that aims to address questions regarding the standardization of national clinical skills assessment after the discontinuation of the Step 2 CS exam. The Clinical Skills Assessment and Standardization (CLASS) Working Group brings together representatives from the American medical education community to “arrive at a meaningful definition of assessment of clinical skills” and to answer important questions regarding the logistics and fairness of such assessments. The CLASS project echoes other calls and ideas for improvements in the way we define, teach, and assess clinical skills [10-12]. One potential approach that has been suggested is the continued formation of regional consortia similar to those currently existing in California, United States, and the Mid-Atlantic, United States [13].

In Florida, United States, representatives from all 10 (eight allopathic and two osteopathic) medical schools have come together to form the Florida Clinical Skills Collaborative (FCSC) to chart a common regional path forward. The highest priority of the FCSC is in line with the larger national agenda of the CLASS working group but has the added advantage of the feasibility of implementation due to scale. Data presented in this manuscript will be presented in poster form at the 2022 AAMC Learn Serve Lead Annual Meeting in Nashville, United States.

## Materials and methods

Members of the FCSC first began meeting informally and virtually in February 2021 after the discontinuation of Step 2 CS/2-PE. Initial attendees included representatives from six allopathic state schools and topics of discussion included COVID-19-related curricular adaptations, issues around simulation-related social distancing and personal protective equipment (PPE), and solutions for assessment challenges. After several meetings, the group formalized, established biweekly Zoom meetings, and invited representatives from all 10 allopathic and osteopathic Florida medical schools (Table 1). In order to develop a shared mental model, we began with brief presentations of the CS assessment program at each institution and established a Microsoft Teams site for document sharing. We then formed subgroups to work on 1) Establishing mission/vision/values statements, 2) Fleshing out logistics/bylaws, and 3) Drafting a proposal for a shared assessment pilot. 

**Table 1:** Florida Clinical Skills Collaborative (FCSC) participating institutions

Institution	Location	Allopathic/Osteopathic	Public/Private
Florida Atlantic University Charles E. Schmidt College of Medicine	Boca Raton	Allopathic	Public
Florida International University Herbert Wertheim College of Medicine	Miami	Allopathic	Public
Florida State University College of Medicine	Tallahassee	Allopathic	Public
Lake Erie College of Medicine	Bradenton	Osteopathic	Private
Nova Southeastern University Dr. Kiran C. Patel College of Allopathic Medicine	Fort Lauderdale	Allopathic	Private
Nova Southeastern University Dr. Kiran C. Patel College of Osteopathic Medicine	Fort Lauderdale	Osteopathic	Private
University of Central Florida College of Medicine	Orlando	Allopathic	Public
University of Florida College of Medicine	Gainesville	Allopathic	Public
University of Miami Miller School of Medicine	Miami	Allopathic	Private
University of South Florida Morsani College of Medicine	Tampa	Allopathic	Public

Our early processes and goals were presented as a Table Topic discussion at the April 2021 meeting of the AAMC Southern Group on Educational Affairs. FCSC members also participated in the September 2021 Directors of Clinical Skills (DOCS) webinar ‘Keys to Successful Clinical Skills Consortia’ and now periodically report updates to the Undergraduate Medical Education Steering Committee for the Florida Council of Deans.

In order to assess satisfaction, explore experienced benefits and challenges, and prioritize goals, an anonymous survey was circulated via Qualtrics in December 2021 to all FCSC members (n=20 individuals representing 10 institutions). Specifically, we assessed, 1) Personal level of interest in joining a regional consortium, 2) Perceived institutional level of interest in joining a regional consortium, 3) Personal satisfaction with membership, and 4) Institutional satisfaction with membership (Likert 1-5). Members were also asked to identify 1) their most important reason(s) for joining the consortium, 2) the most valuable benefit(s) they have received from the membership, 3) the most difficult challenge(s) of membership, and 4) what they think the highest priority of the consortium for the following year (multiple choice with the option to describe "other"). Descriptive statistics (number, mean, and SD) were reported.

## Results

**Figure 1:**
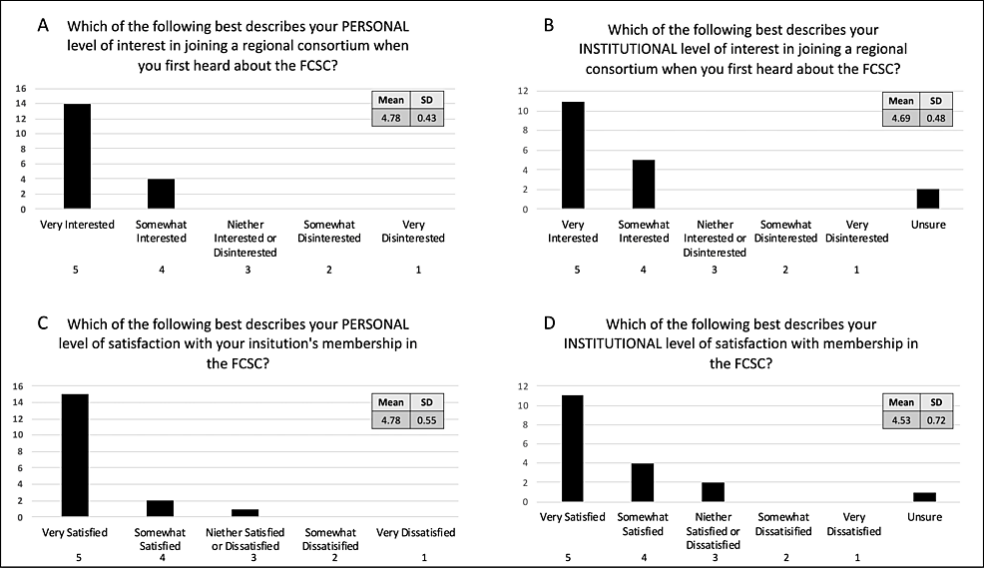
Individual and institutional interest and satisfaction The mean baseline interest in joining the collaboration was found to be 4.78 (SD 0.43) among individuals (A). The mean baseline interest among institutions (as estimated by individual members) was found to be 4.69 (SD 0.48) (B). Individual satisfaction with the decision to join was found to be 4.78 (SD 0.55) (C) and institutional satisfaction was estimated to be 4.53 (SD 0.72) (D). FCSC = Florida Clinical Skills Collaborative

Survey completion was 90% (18/20 individuals representing 10/10 institutions). On a 5-point Likert scale, the mean initial interest in joining the collaboration was found to be 4.78 (SD 0.43) among individuals. Mean interest among institutions (as estimated by individual members) was found to be 4.69 (SD 0.48). Individual satisfaction with the decision to join was found to be 4.78 (SD 0.55) and institutional satisfaction was estimated to be 4.53 (SD 0.72; Figure 1).

**Figure 2:**
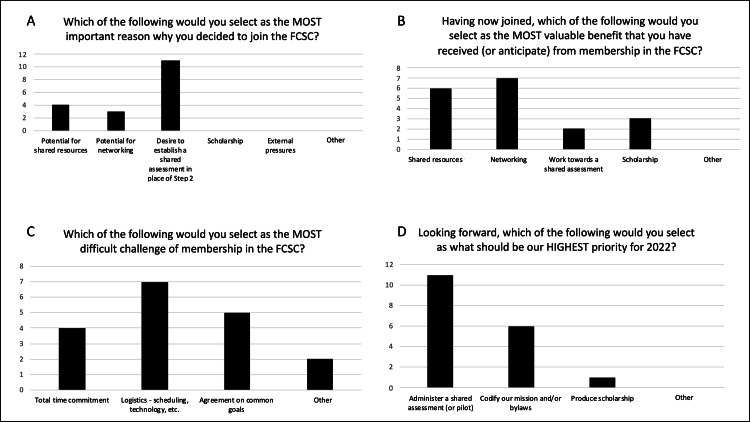
Individuals' reasons for joining, benefits, challenges, and priorities Members most commonly cited “a desire to establish a shared assessment in place of Step 2 CS/2-PE” as the most important reason for joining (A). Experienced benefits of membership were ranked as the following: 1) Networking, 2) Shared resources for curriculum implementation, 3) Scholarship, and 4) Work towards a shared assessment in place of Step 2 CS/2-PE (B). Challenges of membership were ranked as the following: 1) Logistics such as scheduling and technology, 2) Agreement on common goals, 3) Total time commitment, and 4) Other (large group size) (C).  Members cited the “Administration of a Joint Assessment Pilot” as the highest priority for the coming year (D). FCSC = Florida Clinical Skills Collaborative

Members most commonly cited a “desire to establish a shared assessment in place of Step 2 CS/2-PE” as the most important reason for joining. Other important reasons included “potential for shared resources” and “potential for networking.” Experienced benefits of membership were ranked as the following: 1) Networking, 2) Shared resources for curriculum implementation, 3) Scholarship, and 4) Work towards a shared assessment in place of Step 2 CS/2-PE. Challenges of membership were ranked as the following: 1) Logistics such as scheduling and technology, 2) Agreement on common goals, 3) Total time commitment, and 4) Other (large group size). Members cited the “administration of a joint assessment pilot” as the highest priority for the coming year, followed by “codification of the mission statement and/or bylaws” and the “production of scholarship” (Figure 2).

## Discussion

Florida has successfully launched a regional consortium for the assessment of clinical skills competency. Membership includes representation from all 10 Florida medical schools with high levels of both personal and institutional satisfaction. While challenges such as logistics and differing institutional priorities exist, benefits include opportunities for networking, shared resources, and scholarly activity. 

FCSC meetings provide a venue to discuss commonalities and differences in clinical skills teaching and assessment across institutions and have become an important source of support and information for all members. In Florida, regional collaboration has facilitated the exploration of out-of-the-box questions like ‘How do we define CS?’ and more routine questions like ‘What specific skills must we assess prior to graduation?’ Other topics discussed include how assessment drives curricula, how deliberate timing of assessments might better scaffold clinical learning, and the relationship between entrustable professional activities (EPAs) and other assessments. In addition, we have considered how consortia might serve not only as incubators but also as laboratories for new ideas and how defined and trusted regional standards might influence the selection of applicants by residency program directors.

The absence of clinical skills assessment at the national level necessitates even greater responsibility by medical schools to ensure their students’ clinical competence and readiness for supervised practice at the time of graduation. While disruptive, this change will allow medical schools to move away from established structures designed to promote high pass rates and instead to design high-quality assessments better aligned with both national standards and local institutional program objectives.

The FCSC supports broader efforts like the AAMC CLASS Project and the dissemination of resources by national groups, but also aims to facilitate the development of additional regional consortia. Prior projects have shown the potential generalizability of regional exams based on USMLE standards [14,15] and the feasibility of clinical skills testing organized at the regional level both within and outside of the United States [16-18]. The FCSC hopes to contribute to national and international efforts while also helping others to form meaningful collaborations with local and regional partners. 

While this study shows high levels of initial interest and member satisfaction with the decision to join the regional collaboration, limitations include the small study size and its reliance on individual estimations of institutional interest and satisfaction. Future efforts will attempt to address these limitations by collecting satisfaction data from institutional representatives not directly involved in FCSC activities.

## Conclusions

The FCSC illustrates an easily adaptable and generalizable model that has demonstrated feasibility and a high level of satisfaction among participants. In the absence of Step 2 CS/2-PE, medical schools must develop means of ensuring students’ clinical competence and readiness for supervised practice at graduation. The formation of regional consortia may facilitate the design of high-quality assessments better aligned with both national standards and local priorities while also preserving institutional flexibility. Similar to established consortia in California and the Mid-Atlantic, early success of the FCSC suggests the feasibility of regional efforts. The FCSC aims to help others form meaningful collaborations by sharing information on our early processes and challenges. 

It is our belief that overarching recommendations can and should be agreed upon, but that those recommendations should strive to preserve flexibility for implementation and standardization at the local and regional levels. Our collaboration is young, but we have already found ways to grow together. We look forward to continuing our work in Florida while also contributing to ongoing national and international conversations about how best to assess clinical skills.
